# Periodontitis and risk of cancer: Mechanistic evidence

**DOI:** 10.1111/prd.12540

**Published:** 2023-12-15

**Authors:** Giacomo Baima, Margherita Minoli, Dominique S. Michaud, Mario Aimetti, Mariano Sanz, Bruno G. Loos, Mario Romandini

**Affiliations:** ^1^ Department of Surgical Sciences, C.I.R. Dental School University of Turin Turin Italy; ^2^ Department of Periodontology Università Vita‐Salute San Raffaele Milan Italy; ^3^ Department of Public Health and Community Medicine Tufts University School of Medicine Boston Massachusetts USA; ^4^ Faculty of Odontology University Complutense Madrid Spain; ^5^ Department of Periodontology, Faculty of Dentistry University of Oslo Oslo Norway; ^6^ Department of Periodontology, ACTA ‐ Academic Centre for Dentistry Amsterdam University of Amsterdam and Vrije Universiteit Amsterdam Amsterdam The Netherlands

**Keywords:** epidemiology, neoplasms, periodontal disease, periodontal medicine, risk factors, systemic health/disease

## Abstract

This review aims to critically analyze the pathways of interaction and the pathogenic mechanisms linking periodontitis and oral bacteria with the initiation/progression of cancer at different body compartments. A higher risk of head and neck cancer has been consistently associated with periodontitis. This relationship has been explained by the local promotion of dysbiosis, chronic inflammation, immune evasion, and direct (epi)genetic damage to epithelial cells by periodontal pathobionts and their toxins. Epidemiological reports have also studied a possible link between periodontitis and the incidence of other malignancies at distant sites, such as lung, breast, prostate, and digestive tract cancers. Mechanistically, different pathways have been involved, including the induction of a chronic systemic inflammatory state and the spreading of oral pathobionts with carcinogenic potential. Indeed, periodontitis may promote low‐grade systemic inflammation and phenotypic changes in the mononuclear cells, leading to the release of free radicals and cytokines, as well as extracellular matrix degradation, which are all mechanisms involved in carcinogenic and metastatic processes. Moreover, the transient hematogenous spill out or micro‐aspiration/swallowing of periodontal bacteria and their virulence factors (i.e., lipopolysaccharides, fimbriae), may lead to non‐indigenous bacterial colonization of multiple microenvironments. These events may in turn replenish the tumor‐associated microbiome and thus influence the molecular hallmarks of cancer. Particularly, specific strains of oral pathobionts (e.g., *Porphyromonas gingivalis* and *Fusobacterium nucleatum*) may translocate through the hematogenous and enteral routes, being implicated in esophageal, gastric, pancreatic, and colorectal tumorigenesis through the modulation of the gastrointestinal antitumor immune system (i.e., tumor‐infiltrating T cells) and the increased expression of pro‐inflammatory/oncogenic genes. Ultimately, the potential influence of common risk factors, relevant comorbidities, and upstream drivers, such as gerovulnerability to multiple diseases, in explaining the relationship cannot be disregarded. The evidence analyzed here emphasizes the possible relevance of periodontitis in cancer initiation/progression and stimulates future research endeavors.

## 1 INTRODUCTION

Cancer is the second leading cause of death worldwide, accounting for nearly 10 million deaths every year. Around 19 million new cancer cases are diagnosed annually, with breast, lung, colorectal, pancreatic, and gastric cancer being the most common.^1^ Effective prevention and treatment strategies remain elusive, and their effective implementation relies on bridging the gaps on the molecular mechanisms of carcinogenesis and on targeting actionable risk factors.

Cancer has long been defined as a genetic disease, and many incident cancers can be attributed to unmodifiable intrinsic risk, that is, stochastic accumulation of mutations in highly dividing cell populations.^2^ However, a high proportion (estimated 30%–70%) of malignancies is attributable to modifiable risk factors, mainly tobacco smoking, alcohol, high body mass index (BMI), and chronic infections.^3^ Moreover, in recent years, the host microbiome has come under intense scrutiny as a major player in the initiation and progression of neoplasms.^4^ In parallel, chronic local inflammation and its systemic impact have been recognized as the background soil fostering multiple hallmark functions of malignancies.^5^

Periodontitis is a highly prevalent, chronic inflammatory disease resulting in bacterial translocation and low‐grade systemic inflammation (LGSI).^6^ These mechanisms have been argued as the main pathogenic pathways to explain the many epidemiological associations between periodontitis and the most common noncommunicable diseases (NCDs).^7^ Periodontitis and exposure to oral pathobionts have also been linked to increased risk of cancer incidence and mortality in epidemiologic surveys.^8^, ^9^, ^10^ Consequently, a concerted research effort has been devoted to exploring if causation exists beyond association.^11^ This field presents a multi‐scale complexity with significant unresolved caveats. However, it may hold a potential global health impact if the current strategies to prevent and treat periodontitis prove to be beneficial in reducing the burden of malignancies.

After recapitulating the mechanistic hallmarks of the multistep development of human tumors, this critical review explored the complexity of the “periodontal‐hit” cancer interaction in different body regions.

## 2 “MULTI‐HIT” CARCINOGENESIS IN A NUTSHELL

Cancer is a multistep process resulting from complex interactions between endogenous and environmental determinants, and it is usually described as an uncontrolled proliferation of cells that have undergone (epi)genetic alterations.^12^ A mutation at a proto‐oncogene and/or tumor‐suppressor gene—due to stochastic errors in DNA replication or to endogenous/exogenous carcinogens (i.e., tobacco, oxidants)—may confer the cell an intrinsic capacity for out‐of‐control growth. However, a single driver mutation is not sufficient to initiate cancer, since multiple “hits” are usually required.^13^ Subsequently, the initiated cell expands clonally into a detectable mass through a prolonged exposure process called promotion. These ill‐defined preneoplastic masses may subsequently progress to malignant neoplasms by increasing their degree of heterogeneity and invasiveness.^14^

To evade the host antitumor checkpoints, the mutated cells need to acquire a certain set of “hallmarks,” such as the ability to sustain proliferative signaling, reprogramming of epigenetic regulation and energy metabolism, evade growth suppressants, resist cell death and immune destruction, unlock unlimited replicative potential and phenotypic plasticity, induce angiogenesis, sustain invasion, and metastasis.^15^ At the background, inflammation—the “enabling characteristic”—favors multiple of those hallmark functions in the local niche through hypoxia, acidosis, and reactive oxygen species (ROS).^5^ In this context, the role of the “tumor microenvironment” has acquired relevance in the contribution to tumorigenesis, fostering the aberrant traits of cancer cells.^16^ These microenvironmental conditions can change due to aging, comorbidities, and molecules released by the host‐associated polymorphic microbiome.^5^, ^17^

## 3 THE CANCER‐ASSOCIATED MICROBIOME AND THE ORAL RESERVOIR

The role of microorganisms as tumor promoters in specific cancers has been long recognized, with infectious agents contributing upward of 15%–20% of malignancies. Indeed, *Helicobacter pylori* in gastric cancers, human papillomavirus (HPV) in cervical and head/neck cancers, and hepatitis B/C virus (HBV/HCV) in hepatocellular carcinomas are well‐established carcinogens.^3^ Advances in next‐generation sequencing have expanded this concept, suggesting that most human cancer types may harbor specific microbiota signatures, bringing a high potential for new diagnostic and therapeutic approaches.^4^

While the gut has been considered the main source of this tumor‐associated microbiota, recent research has focused on integrating the oral and the gut microbiome as a whole (i.e., from mouth to rectum).^5^ The oral cavity is indeed a complex microbial ecosystem at the entry point to the human body, containing up to 2000 bacterial, archaeal, viral, and fungal species, many of which are noncultivable.^18^ Most oral microorganisms are commensals, although some opportunistic inflammophilic pathobionts possessing oncogenic properties may emerge. Besides their detrimental effect at the levels of teeth and gum,^19^ members of this meta‐organism (i.e., the oral microbiome) may translocate to multiple body districts, especially in periodontitis as a consequence of the compromised mucosal barrier and the high dysbiotic bacterial load within pockets and saliva.^20^

**FIGURE 1:**
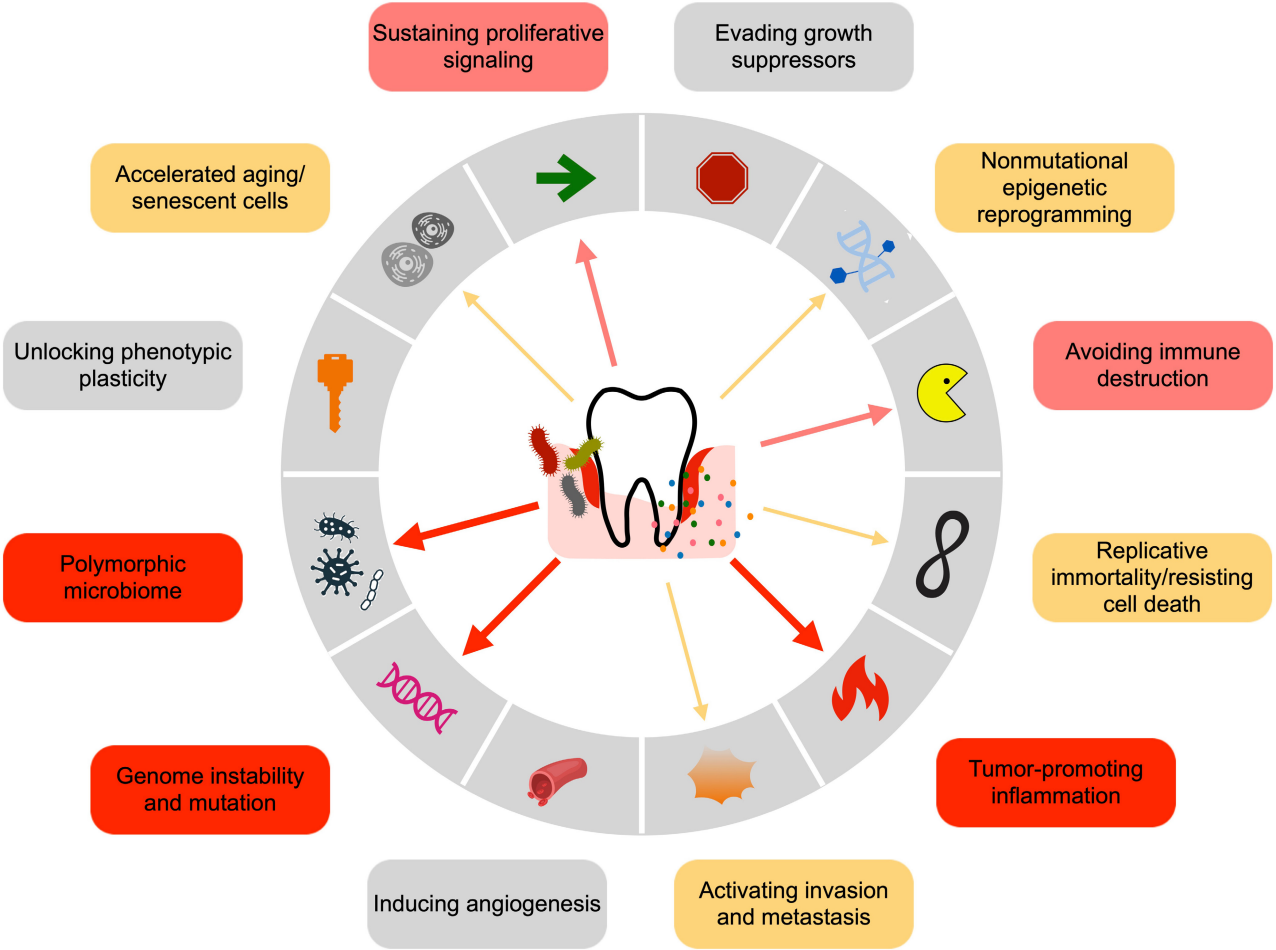
The proposed routes for a periodontitis signature in relation to the cancer hallmarks and enabling characteristics, described by Hanahan (2022). The presence and thickness of the arrows reflect the available amount of evidence.

**FIGURE 2:**
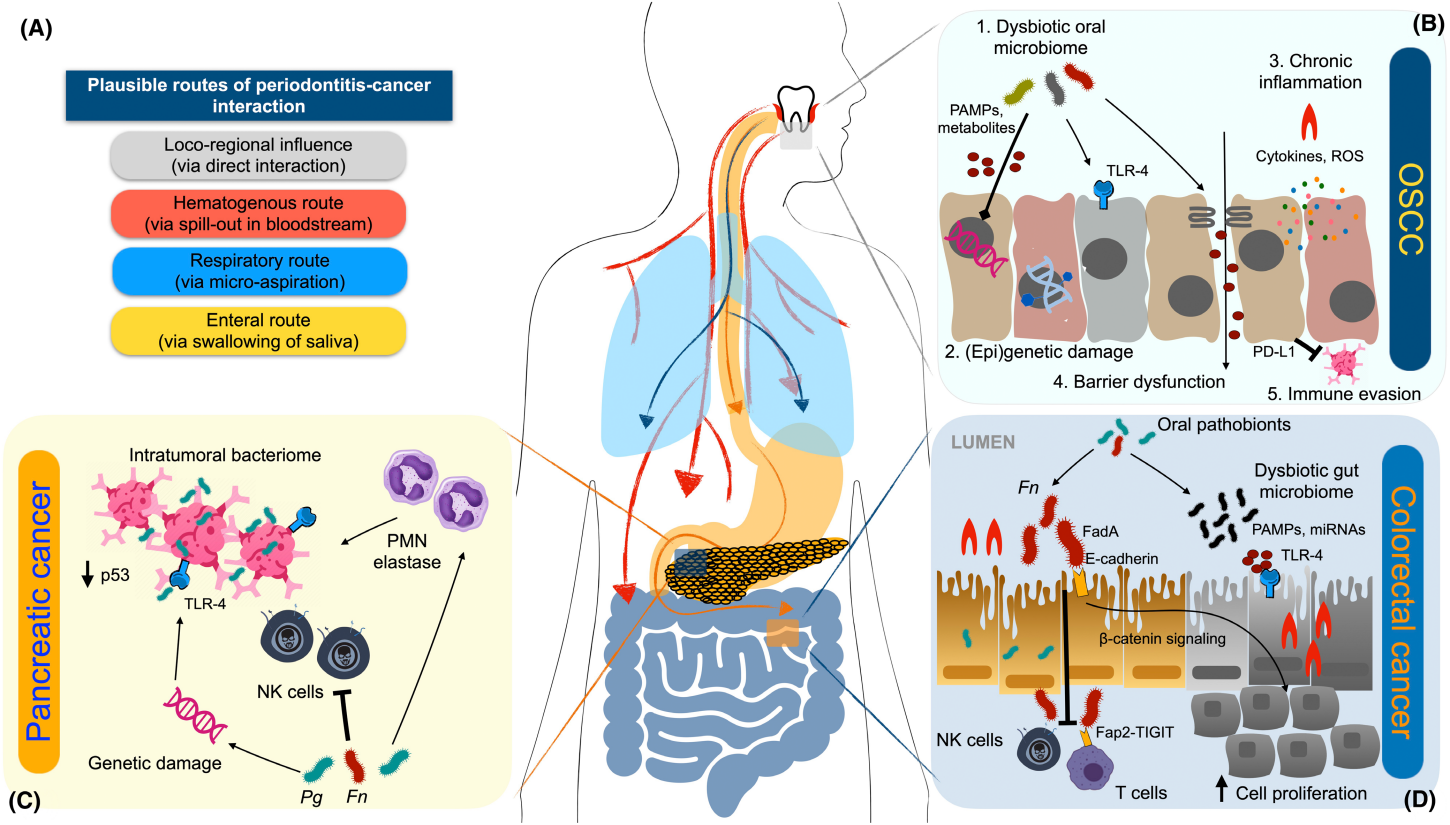
(A) Proposed principal routes of periodontal inflammation and oral bacteria in cancer development. (B) In head and neck cancer, periodontal bacteria act by: 1. direct action of virulence factors; 2. epigenetic and gene expression modulation; 3. chronic inflammation via pathogen‐associated molecular patterns (PAMPs) and reactive oxygen species (ROS); 4. epithelial barrier dysfunction; and 5. immune evasion. (C) In pancreatic cancer, *Fusobacterium nucleatum* (*Fn*) and *Porphyromonas gingivalis* (*Pg*) may induce genetic damage and reduction of p53 triggering antiapoptotic characteristics within the intratumoral bacteriome, whereas their lipopolysaccharide may activate TLR‐4 and NF‐κB signaling, promoting inflammation. These bacteria also exert immune evasive effects on natural killer (NK) cells and promote elastase release from tumor‐associated polymorphonuclear cells (PMN). (D) In colorectal cancer, *Fn* may promote cell proliferation via FadA adhesin‐E‐cadherin interaction, a pro‐carcinogenic environment via miRNA‐mediated activation of TLR2/TLR4, and immune evasion by suppressing NK and effector T cells via the Fap2‐T‐cell immunoglobulin and ITIM domain (TIGIT) interaction.

Being such a repository, the inflamed periodontal tissues may impact on the development of various chronic extra‐oral comorbidities, including cardiovascular diseases, diabetes mellitus, and tissue‐specific cancers too.^21^, ^22^, ^23^, ^24^, ^25^, ^26^ Indeed, translocating periodontal pathobionts and hematogenous spilling‐out of pro‐inflammatory mediators from the subgingival microenvironment can also interact with the fundamental hallmarks of carcinogenesis (Figures 1 and 2A).

## 4 THE LOCOREGIONAL INFLUENCE: HEAD AND NECK CANCERS

Epidemiological studies have consistently pointed out to an increased risk of head and neck cancer (HNC) in periodontitis subjects (Table S1). A large prospective cohort study on almost 20 000 never‐smoker US men reported a 2.3‐fold increase in the risk of oropharyngeal cancer among subjects with self‐reported periodontitis.^27^ A case–control study with a comparable sample size also revealed an increased risk of HNC among subjects with a history of periodontitis, in a multinational population without restrictions in terms of gender and smoking status.^28^ These findings were consistent with another case–control study in South Korea, which reported an increased risk of oral squamous cell carcinoma (OSCC) in radiographically diagnosed periodontitis subjects.^29^

The analysis of OSCC histological samples has indicated the presence of periodontitis‐associated microorganisms, including *Fusobacterium*, *Peptostreptococcus*, *Filifactor*, *Catonella*, and *Parvimonas* taxa. In fact, *Porphyromonas gingivalis* and *Fusobacterium nucleatum* have been detected up to 600 times more frequently in OSCC than in para‐cancerous and normal tissues.^30^ Although these findings can also be secondary to bacterial colonization of an already existing lesion, the possible role of periodontal bacterial dysbiosis as inducer of HNC can be explained by at least five mechanisms (Figure 2B).^31^

First, the altered microbiome can directly contribute to carcinogenesis through its toxins and metabolites since they possess the ability to induce genetic damage in the epithelial cells. For example, *Aggregatibacter actinomycetemcomitans* secreted cytolethal distending toxin and other byproducts, such as ROS, nitrogen reactive species, sulfides, nitrosamines, and acetaldehyde have shown oncogenic potential through DNA alkylation, mutations, and impaired repair.^32^, ^33^

Second, certain oral pathobionts can induce epigenetic changes by gene expression regulation. In a recent preliminary analysis based on DNA methylation patterns in oral brushing specimens, some inflammatory genes (GP1BB, MIR193, and ZAP70) were found differentially regulated between OSCC patients, periodontitis patients, and healthy volunteers.^34^ Also, *F. nucleatum* and *P. gingivalis* have shown to downregulate p53 and Ku70 pathways through histone H2A phosphorylation, thus promoting increased cell proliferation of OSCC cells up to 125 times.^35^ In addition, a recent meta‐transcriptomic analysis revealed an increased number of *F. nucleatum*‐related transcripts at tumor sites compared to healthy controls.^36^ Also, *P. gingivalis* membrane W83 component possesses the ability to alter transcriptomic readouts in OSCC cells, mainly belonging to pathways related to cell metabolism, inflammation, and increased replication.^37^

A third pathway may involve the tumor‐promoting inflammation induced by the periodontitis‐associated microbiome, which may lead to an increase in cell proliferation/migration and inhibition of apoptosis. In this respect, toll‐like receptors (TLRs) are critical in the microbiome/immunity interplay, transducing the proinflammatory stimuli from pathogen‐associated molecular patterns (PAMPs) through nuclear factor (NF)‐κB activation.^38^ Mice constitutively expressing TLR4, a lipopolysaccharide (LPS) ligand, display an increased epithelial tumorigenesis, while TLR4‐deficient mice are somehow protected.^39^ Also, *P. gingivalis* and *F. nucleatum* can upregulate the genes involved in downstream TLR, NFκB, and MAPK signaling pathways in both normal and malignant oral epithelial cells.^40^, ^41^

The fourth circuit involves epithelial barrier function impairment, through microbial PAMPs and metabolites (e.g., lactic, acetic, butyric, and isobutyric acids), which in turn may enhance the carcinogenic pathways.^42^ For example, *P. gingivalis* and *Treponema denticola* can degrade gingival epithelial junctional adhesion molecule‐1, zonula occludens‐1, and occludin.^43^ However, this increased permeability may also support the hypothesis of a secondary colonization of HNC tissues by pathobionts, without any causal role.

Lastly, periodontal pathobionts can induce immune evasion locoregionally by modulating the expression of critical immune checkpoints. B7‐H1 and B7‐DC, ligands of programmed death receptor (PD‐L1), regulate immune response and are highly expressed in OSCC, enabling tumor cells to evade host immunity by suppressing activated T‐cell functions and survival.^44^ Consistent evidence has shown that *P. gingivalis* strains are able to upregulate B7‐H1 and B7‐DC receptors in OSCC, thereby facilitating immune evasion and tumor progression.^45^, ^46^, ^47^

In addition to these five circuits involving periodontal bacteria, periodontitis may also facilitate the acquisition and persistence of oral HPV, which accounts for up to 25% of HNC cases. Indeed, chronic periodontal inflammation increases the exposed epithelial area and the expression of cell‐surface heparan sulphate, one of the HPV receptors.^48^ Accordingly, a history of periodontitis has been associated with HPV‐positive HNC cases.^49^, ^50^

All these mechanisms support the evidence of a polymicrobial dysbiosis‐associated carcinogenesis model to explain the association between periodontitis and HNC, where the synergic interaction of microbial communities, including the virome and the mycobiome, may induce the oncogenic potential. In this respect, data from transcriptomic and metabolomic studies are strengthening the tenet that functional properties of the oral microbiome have more relevance to cancer development than strict taxonomic variations.^51^, ^52^

## 5 THE ECTOPIC COLONIZERS: ORAL PATHOBIONTS IN LUNG, BREAST, AND PROSTATE CANCER

Tables S2–S4 displays the most relevant epidemiological studies linking periodontitis with lung, breast, and prostate cancers; however, this scientific evidence is far from being conclusive.

### 5.1 Lung cancer

The cohort study by Michaud et al.^27^ failed to report an independent association between self‐reported periodontitis and lung cancer in never‐smoker US males. However, another cohort study in USA with clinical evaluation of the periodontal status, reported an increased risk of lung cancer in severe periodontitis subjects (adjusted hazard ratio (aHR) = 2.37, 95% CI: 1.41–3.99).^53^ A recent systematic review of cohort studies confirmed these findings, although with slightly lower estimates (adjusted relative risk (aRR) = 1.40, 95% CI: 1.25–1.58)^54^ (Table S2).

There is emerging evidence on the important role of the microbiome in lung cancer, with the presence of *Acinetobacter*, *Pseudomonas*, *Prevotella*, *Veillonella*, and *Streptococcus* genera in the tumor microenvironment.^55^ These microbial species of oral origin can migrate to the lungs via micro‐aspiration, and may contribute to chronic obstructive pulmonary disease, pneumonia, and even increased severity of COVID‐19.^56^ Indeed, patients with squamous cell and adenocarcinoma displayed an altered salivary microbiota composition compared to healthy subjects, particularly of the genera *Capnocytophaga* and *Veillonella*.^31^ Liu et al.^57^ also reported a higher presence of *P. gingivalis* in carcinoma‐associated than in adjacent lung tissues. Notably, the survival rate of patients with *P. gingivalis* colonization in the same cohort was significantly shortened. Nevertheless, data on the impact of oral bacteria on lung cancer pathogenesis remain limited, and more studies with improved collection methods are needed. Again, these oral pathogens may also be regarded as secondary colonizers, probably due to the so‐called “enhanced permeability and retention effect.”^58^ Indeed, circulating microbes and macromolecules leak preferentially into cancer tissue and may be then retained into the tumor bed, due to enhanced permeability of the tumor vasculature and reduced lymphatic drainage.^59^

### 5.2 Breast cancer

A large cohort study in the USA consisting of 65 869 postmenopausal women reported an incidence of 7149 cancer cases over a mean follow‐up of 8.3 years. For women with self‐reported periodontitis, an increased risk of breast cancer was found (aHR = 1.13, 95% CI: 1.03–1.23).^60^ The study by Michaud et al.^53^ failed to identify a significant association when employing a clinical examination protocol for assessing periodontitis. However, a systematic review including a total of 3538 breast cancer cases out of 168 111 participants, reported an increased risk of breast cancer in women affected by periodontitis (aRR = 1.18, 95% CI: 1.11–1.26)^61^ (Table S3).

Cancerous human breast tissues have also demonstrated presence of oral bacteria, particularly *F. nucleatum*.^62^ Consequently, Parhi et al.^63^ recently tested whether a periodontal pathobiont‐induced bacteremia could contribute to breast tumorigenesis in mice. Intravascularly inoculated *F. nucleatum* colonized breast tissue using its lectin Fap2, which attached to tumor‐expressed Gal‐GalNAc with a 100‐fold increase in abundance compared with normal tissues. Once in situ, *F. nucleatum* suppressed the accumulation of tumor‐infiltrating T cells, and promoted tumor growth and metastatic progression, which were counteracted by antibiotic treatment. It was also shown that Gal‐GalNAc levels increased as human breast cancer progressed, correlating with *F. nucleatum* DNA detection.^31^

### 5.3 Prostate cancer

Table S4 displays the most relevant epidemiological studies linking periodontitis with prostate cancer, indicating conflicting results. In the study by Michaud et al.,^27^ self‐reported periodontitis was not associated with an increased risk of prostate cancer in never‐smoker males. The results were consistent in a large US cohort including clinical measures of periodontal disease.^53^ Conversely, a large cohort study with 2063 cases of >120 000 South Koreans reported a significant association between clinically assessed periodontitis and prostate cancer (aHR = 1.24, 95% CI: 1.16–1.32).^64^

Interestingly, oral inflammophilic bacteria (i.e., *P. intermedia*, *P. gingivalis*, and *T. denticola*) were found in prostatic secretions of patients with both chronic prostatitis and periodontitis.^65^ It may be hypothesized that, once colonizing the prostate through bacteremia or bladder infection, these bacteria may lead to a local chronic inflammatory response, which in turn may accelerate prostate cancer onset and progression. Moreover, *P. gingivalis* may upregulate the signaling pathway of PD‐L1, leading to immune evasion in the prostate tumor environment.^66^ Nevertheless, both the epidemiological evidence and possible pathways linking the periodontitis to prostate tumorigenesis are still insufficient, and therefore, further studies are needed.

## 6 THE ENTERAL WAY: PERIODONTAL PATHOBIONTS AND GASTROINTESTINAL CANCERS

Tables S5–S7 display the most relevant epidemiological evidence linking periodontitis with gastrointestinal cancers, reporting consistent positive associations for the more proximal anatomical locations (esophageal/gastric and pancreatic cancers), and conflicting evidence for the more distal sites (colorectal cancer).^67^

### 6.1 Esophageal and gastric cancer

In the cohort study by Nwizu et al.,^60^ a positive association (aHR = 3.28, 95% CI: 1.64–6.53) between self‐reported periodontitis and incidence of esophageal cancer was observed in postmenopausal women. Consistently, another cohort study also including men reported a significant association, albeit with a lower estimate (aHR = 1.43, 95% CI: 1.05–1.96).^68^ This same study also reported a positive association of self‐reported periodontitis with incidence of gastric cancer (aHR = 1.52, 95% CI: 1.13–2.04). Similarly, a large‐scale study in South Korea with 3920 cases among >700 000 subjects, found a modest, albeit significant association between clinically assessed periodontitis and gastric cancer (aHR = 1.14, 95% CI: 1.04–1.24)^69^ (Table S5).

With regard to the mechanisms, the evidence is more limited, although *P. gingivalis* was detected in 61% of esophageal squamous cell carcinoma tissues, 12% of adjacent tissues, and never in the healthy mucosa.^70^ Similarly, *F. nucleatum* was recently associated with lower long interspersed nuclear element‐1 DNA methylation and worse prognosis in diffuse‐type gastric cancers.^71^ An alternative pathway is related to the possible role of periodontal pockets as reservoirs for *H. pylori*—the only bacterium classified to date as carcinogenic.^72^ Indeed, a recent epidemiological study indicated an association between periodontitis and mortality due to gastrointestinal cancers only in subjects with *H. pylori* seropositivity.^73^

### 6.2 Pancreatic cancer

A prospective US‐based cohort study on 51 529 males (216 cases) aged 40–75 years pointed out to an increased incidence of pancreatic cancer in subjects self‐reporting a history of periodontitis (aRR = 1.64, 95% CI: 1.19–2.26).^74^ Data from a systematic review with 653 cases among >300 000 subjects with no restrictions in terms of gender and age‐reported comparable results (aRR = 1.74, 95% CI: 1.41–2.15)^75^ (Table S6).

The etiology of pancreatic cancer is largely unknown. Together with smoking, chronic pancreatitis, and overweight/obesity, local microbial dysbiosis has been accounted as a plausible risk factor.^3^, ^76^ Presence of a specific intratumoral microbiome has been documented in pancreatic cancers, and its perturbation by antibiotic therapy restored the immune system activity through an antitumor Th1 response.^77^ Moreover, invasive adenocarcinomas and pancreatic cysts may be enriched in members of the oral microbiome. Indeed, high‐grade dysplasia cysts presented an increased *F. nucleatum* and *Granulicatella adiacens* load compared to low‐grade cysts, while *F. nucleatum*‐positive pancreatic ductal adenocarcinomas have been associated with higher mortality.^78^ Also, *T. denticola* dentilisin, a virulence factor with strong surface protease activity, was detected in 65% of the pancreatic cancer tissues.^79^ Similarly, two large prospective cohort studies reported that oral carriage of *P. gingivalis* and *A. actinomycetemcomitans*,^80^ as well as high levels of antibody titers against five periodontal pathogens,^24^ were associated with an increased incidence of pancreatic cancer. These observations were recently replicated in mice in a subcutaneous pancreatic model, where tumor development was induced after *P. gingivalis* gavages.^81^ Mechanistically, *P. gingivalis* and *F. nucleatum* possess broad immune evasive effect on natural killer (NK) cells and antiapoptotic characteristics, as well as the ability to invade pancreatic cells, which in turn may cause genetic damage and reduction of p53 tumor suppressor levels. Also, their LPS may accelerate carcinogenesis via TLR4 binding and NF‐κB signaling, boosting the inflammasome (the cytosolic oligomers involved in the activation of innate immunity) and polymorphonuclear (PMN) elastase release from tumor‐associated neutrophils (Figure 2C).^82^

### 6.3 Colorectal cancer (CRC)

Among never‐smoker US males, Michaud et al.^27^ did not find an association between increased risk of CRC and self‐reported periodontitis. The same group, however, reported a positive association (aHR = 1.51, 95% CI: 0.90–2.52), albeit not statistically significant, in a different US cohort including both smokers and nonsmokers and both males and females, and employing a clinical examination to identify severe periodontitis cases.^53^ Recently, a population‐based cohort study found that periodontitis may carry an increased risk of developing benign, but not malignant, colorectal tumors.^83^ A systematic review of cohort studies reported a similar estimate of association (aRR = 1.44, 95% CI: 1.18–1.76).^84^ Consistently, a large cohort study with 3416 cases of more than 700 000 South Koreans reported a significant, albeit modest association between clinically assessed periodontitis and CRC (aHR = 1.13, 95% CI: 1.03–1.24)^69^ (Table S7).

Recent next generation sequencing studies have revealed that the microbiomes associated with distal digestive tract cancers contain bacterial species plausibly originating from the oral cavity.^85^ Specifically, *F. nucleatum* has been identified among the most prevalent bacterial species in CRC tissues. Although the evidence for its translocation from the oral environment to the gut needs to be confirmed by strain‐level metagenomics techniques, the mechanistic role of *F. nucleatum* as the “alpha‐bug” with direct tumor‐accelerating features has been repeatedly suggested.^86^, ^87^ Briefly, enhancement of cancer cell proliferation, establishment of a tumor‐promoting immune environment through miRNA‐mediated activation of TLR2/TLR4, and the evasion of immune checkpoints have been proposed as its main mechanisms.^88^ In mice intestine, *F. nucleatum* was indeed shown to promote tumor progression by suppressing NK and effector T cells, via the Fap2‐T‐cell immunoglobulin and ITIM domain (TIGIT) interaction.^89^ Another potential *F. nucleatum* involvement in CRC has been recently unveiled, since it can modulate the cadherin/β‐catenin signaling and annexin A1 expression. Through its unique FadA adhesin binding to E‐cadherin on the surface of colonic epithelial cells, *F. nucleatum* can also activate β‐catenin signaling and as such increase the expression of oncogenic and inflammatory genes in CRC cells (Figure 2D).^90^ Complementary with the enteral way, a hematogenous route for *F. nucleatum* to reach colon adenocarcinomas has been tested in mice. As was shown in breast tumor models, *F. nucleatum* binding to tumor cells seems to occur by Fap2 mediated recognition of Gal‐GalNAc.^91^ Beside this extensively studied pathobiont, also *P. gingivalis* can negatively interact with colon carcinoma cells, possibly upregulating the inflammatory pathway pivoted by PD‐L1 as described also for OSCC and prostate cancer.^92^

Despite this proposed carcinogenic role by specific oral pathogens, the possible role of periodontitis‐derived systemic inflammation through dissemination of immunological components has also accumulated recently. In fact, activated T‐helper 17 lymphocytes from periodontal inflammatory lesions, may translocate to the gastrointestinal tract and contribute to gut microbial dysbiosis and inflammation.^93^ This recently discovered immunological pathway deserves further experimental validation in light of the relevance that these cell populations play in gut mucosal carcinogenesis.^94^

## 7 PERIODONTAL IMMUNE DISTURBANCES AND MALADAPTIVE HEMATOPOIESIS: THE LINK WITH NONSOLID MALIGNANCIES

The potential epidemiologic link between periodontitis and nonsolid malignancies is displayed in Table S8. In a cohort study from the USA with 1341 cases among more than 50 000 males, self‐reported periodontitis was associated with an increased risk of non‐Hodgkin lymphomas (aHR = 1.26, 95% CI: 1.06–1.49) and chronic lymphocytic leukemia/small lymphocytic lymphomas (aHR = 1.41, 95% CI: 1.04–1.90).^95^ Similarly, the cohort study by Kim et al.^69^ with 399 cases among >700 000 South Koreans, reported that clinically assessed periodontitis was also associated with leukemia (aHR = 1.39, 95% CI: 1.04–1.87). The same study, however, failed to document a positive association with Hodgkin and non‐Hodgkin lymphomas, as well as with multiple myeloma.^69^ Similarly, two cohort studies with US participants described nonsignificant associations between periodontitis and nonsolid malignancies.^53^, ^60^

An emerging field of research is focusing on the possibility that periodontitis may epigenetically prime the hematopoietic progenitors in the bone marrow toward an exacerbated innate immune response.^96^ This excessive recruitment and “dysregulated” priming of the PMNs and peripheral blood mononuclear cells (PBMC) may be implicated in a wide range of chronic inflammatory diseases, including cancers.^20^, ^97^ For instance, Carvalho‐Filho et al.^98^ found that upon the exposure to the *P. gingivalis* protein HmuY, PBMCs from patients with periodontitis downregulated genes associated with apoptosis and other cancer hallmarks. However, the available epidemiologic evidence and precise mechanisms governing this periodontal‐bone marrow axis in nonsolid tumors are still inconclusive and further studies are warranted.

## 8 PERIODONTAL MICROORGANISMS, EPITHELIAL–MESENCHYMAL TRANSITION, AND METASTASIZATION

Only the South‐Korean cohort study by Kim et al.^69^ evaluated the association between periodontitis and incidence of secondary cancer. Even though incidence rates were slightly higher in patients with periodontitis (aRR = 1.15, 95% CI: 0.81–1.65), the reported association was not statistically significant. However, a survival bias cannot be ruled out, since in this study only surviving patients at 5 years after primary malignancy diagnosis were included, whereas periodontitis patients are associated with a higher cancer mortality (Table S9).^9^

Epithelial–mesenchymal transition (EMT) is one of the key developmental mechanisms implicated in the ability of transformed epithelial cells to invade and disseminate.^99^ *F. nucleatum* and *P. gingivalis* have shown to significantly upregulate Snail protein and mesenchymal markers (vimentin), as well as epithelial markers (E‐cadherin) in OSCC cells in vitro. *P. gingivalis* also induced nuclear localization of ZEB1 (zinc finger E‐box‐binding homeobox 1) transcription factor, which controls EMT; interestingly, *P. gingivalis* strains lacking FimA showed less ability.^100^ *P. gingivalis* can also upregulate the expression of ZEB2 via pathways involving β‐catenin and FOXO1 (forkhead box O1).^101^ Recently, periodontal inflammation promoted metastasis of breast cancer by recruiting myeloid‐derived suppressor cells in a murine model.^102^ Induction of pyroptosis by IL‐1β generation and downstream CCL2, CCL5, and CXCL5 signaling were the principal mechanisms involved.

## 9 CAUSALITY AND CASUALTY: COMMON RISK FACTORS AND BACKGROUND ENTITIES

Although these presented hypotheses are tempting, the research findings obtained so far can only scratch the surface of the real complexity of the periodontitis–cancer association. The multistep chronic nonlinear process of carcinogenesis limits the possibility to individuate low‐hanging mechanistic insights in humans. Moreover, longitudinal associations do not necessarily guarantee causality. Also, the strength of the findings from the existing investigations is hampered by the heterogeneity of the methods used to measure the exposure, the study designs, the platforms employed for microbial analyses, the nature of the biological samples, and the risk of publication bias.^11^

Moreover, the possibility that the periodontitis–cancer epidemiologic link may be confounded by residual common risk factors and comorbidities,^103^, ^104^, ^105^, ^106^ as well as background entities should not be underestimated. It could be indeed considered that periodontitis is also a surrogate marker for low socioeconomic status and unfavorable lifestyle behaviors,^107^, ^108^ which constitute well‐established risk factors for certain cancers. A robust bidirectional relationship between periodontitis and metabolic/inflammatory diseases, such as diabetes mellitus and obesity, is also well acknowledged.^109^, ^110^ These comorbidities may potentially contribute in mediating the effect of periodontitis on cancers. Indeed, periodontal treatment proved effective in reducing most of cardiometabolic markers and it can be potentially considered as an arrow in the cancer preventive strategy.^20^, ^111^

Interestingly, multimorbidity has shown to be more prevalent among patients with periodontitis.^112^ This may be ascribed to a background entity, which may both assume socioeconomic and biological connotates. Indeed, while the concept of “syndemics” may support the former (i.e., populations deprived of resources and living in poor conditions, suffering more often from diseases and die earlier/younger), the so‐called systemic effect of periodontitis might be also considered as a result of an upstream gerovulnerability to multiple diseases, pivoted on immune senescence and accelerated biological aging.^113^, ^114^ Finding a way to target this shared, underlying pathology at the interaction between periodontitis and LGSI‐related diseases could be key in better managing patients and reducing the associated economic burden.

## 10 CONCLUDING REMARKS AND FUTURE RESEARCH PRIORITIES

Despite the limitations outlined in this article, there is a general emerging indication that the presence of periodontitis and its associated dysbiosis may contribute to carcinogenesis or tumor progression, especially for HNC and upper digestive tract cancers (Table 1). The main contributing mechanisms to carcinogenesis may involve triggering inflammation (the enabling characteristic), immune system dysregulation, and microbial dysbiosis. Specifically, tumor‐associated microbiomes have been identified, often with periodontal pathobionts being habitual residents.

**TABLE 1:** Periodontitis and cancer: Summary of epidemiologic and mechanistic evidence.

Cancer type	Epidemiologic evidence	Oral pathogens in the intratumoral microbiome	Mechanistic evidence
Head and neck cancer	Positive association, consistent among several studies, especially for OSCC	OSCC samples enriched for *Fusobacterium*, *Filifactor, Peptostreptococcus*, and *Catonella*. *Pg* and *Fn* detected 600 times more in OSCC than normal tissue.	Periodontal bacteria directly exert: Carcinogenic damage by virulence factors Epigenetic and gene expression modulation Chronic inflammation via PAMPs/ROS Epithelial barrier dysfunction Immune evasion Periodontal inflammation increases HPV receptors
Lung cancer	Possible positive association, but evidence is not conclusive	Enrichment of oral genera *Acinetobacter*, *Prevotella*, and *Veillonella*	Little mechanistic evidence available, mainly involving periodontal pathogen (micro)aspiration
Breast cancer	Possible positive association, but evidence is not conclusive	Consistent detection of *Fn* DNA in tumor bacteriome	Hematogenous colonization of *Fn* via lectin Fap2 interaction with Gal‐GalNAc
Prostate cancer	Possible lack of association, but evidence is not conclusive	*Pi*, *Pg*, and *Td* DNA in prostatic secretions	*Pg* upregulation of programmed death ligand 1 pathway
Esophageal and gastric cancer	Positive association, consistent among several studies	*Pg* detected in esophageal cancer tissues, *Fn* in gastric	Periodontal pockets reservoir for *H. pylori* *Fn*‐mediated DNA methylation
Pancreatic cancer	Positive association, consistent among several studies	*Fn* and *Td* detected in adenocarcinomas	*Fn* and *Pg* cell invasion, genetic damage and reduction of p53 *Fn* and *Pg* immune evasive properties: TLRs and complement system manipulation; inhibition of IL‐8 production; degradation of secreted cytokines LPS activates TLR‐4 and NF‐κB signaling, promoting inflammation
Colorectal cancer	Possible positive association, but evidence is not conclusive	*Fn, Peptostreptococcus*, *Leptotrichia*, and *Campylobacter* overabundant in CRC	*Fn* promotes: cell proliferation via FadA adhesin‐E‐cadherin interaction pro‐carcinogenic immune environment via miRNA‐mediated activation of TLR2/TLR4 immune evasion by suppressing cytotoxic and effector T cells
Nonsolid malignancies	Not enough evidence	Not enough evidence	Maladaptive trained immunity and clonal hematopoiesis
Metastasis	Not enough evidence	Not enough evidence	Induction of EMT in epithelial cells by *Fn* and *Pg* via upregulation of Snail protein, vimentin, and ZEB1‐2

Abbreviations: CRC, colorectal cancer; EMT, epithelial–mesenchymal transition; *Fn*, *Fusobacterium nucleatum*; HPV, human papilloma virus; OSCC, oral squamous cell carcinoma; PAMPs, pathogen‐associated molecular patterns; *Pg*, *Porphyromonas gingivalis*; ROS; reactive oxygen species; *Td*, *Treponema denticola*; TLR, toll‐like receptor; ZEB, zinc finger E‐box‐binding homeobox.

Notably, while the epidemiologic evidence is robust for certain types of cancers, more data are warranted for many others, including nonsolid, breast and prostate tumors. Also, much mechanistic research is needed to disentangle the complex, multifactorial interactions that result in disease, and clarify whether periodontal inflammation may directly promote carcinogenesis in the tumor microenvironment, or simply contribute to the systemic inflammatory milieu on which the multistage process of carcinogenesis develops. Moreover, much emphasis has been placed on *P. gingivalis* and *F. nucleatum*, while an exciting potential for future generations of researchers resides in exploring the mechanistic contributions of periodontitis‐related noncultivable bacteria, viruses, fungi, *Entamoebas*, as well as the microbiome‐as‐a‐whole. Murine models of cancer development with experimentally induced periodontitis, large prospective cohort studies including gold‐standard exposure ascertainment and longitudinal collection of tissue and microbial samples, as well as interventional studies, are needed. Advanced molecular technologies, such as gene expression profiling, strain level resolution metagenomics, metabolomics, microRNAs, gene discovery, and pathway analysis will also elucidate the potential role of oral pathobionts as therapeutic targets or diagnostic markers.

The burden of cancer remains one of the most important public health challenges, with modifiable risk factors being pivotal contributors to cancer development and mortality. This critical review highlighted the need for future research commitments projecting beyond the known mechanisms, as well as policies aimed at reducing exposure to risk factors from the oral cavity as part of comprehensive cancer preventive efforts.

## AUTHOR CONTRIBUTION

G. Baima contributed to study conception and design, data acquisition and interpretation, and article drafting; M. Minoli contributed to data acquisition and interpretation, and article drafting; D.S. Michaud, M. Aimetti, B.G. Loos, and M. Sanz contributed to data interpretation, and critically revised the article; M. Romandini, contributed to study conception and design, data acquisition and interpretation, and article drafting. All authors gave final approval and agreed to be accountable for all aspects of the work.

## CONFLICT OF INTEREST STATEMENT

The authors declare no conflicts of interest related to this study.

## Supporting information


Appendix S1.

## Data Availability

No new data was generated, since this is an invited narrative review.
