# Supplementary material for: Periodontitis and risk of cancer: Mechanistic evidence
Source: Periodontol 2000. 2023 Dec 15;96(1):83–94. doi: 10.1111/prd.12540 (PMC11579815; doi:10.1111/prd.12540)
Supplement: Supplementary file 1 — Appendix S1. [file PRD-96-83-s001.docx]

**Supplementary Table 1**. Epidemiological evidence linking periodontitis to head and neck cancer

| **Head and neck cancer** | | | | | | | | |
| --- | --- | --- | --- | --- | --- | --- | --- | --- |
| **Reference** | **Country** | **Study design** | **Length of follow-up** | **Main sample characteristics** | **Assessment of periodontal status** | **Exposure and comparison** | **Cancer assessment** | **Association with cancer** |
| Hashim et al. (2016) | Multinational | Case-control | NR | 21,452 subjects | Self-reported | **Exposure:**  Gum disease (periodontitis or gingivitis)  **Comparison:**  All the remaining participants | Medical records | **Head and neck cancer:**  aOR=1.06 (95% CI: 1.01-1.12) |
| Michaud et al. (2016) | USA | Prospective Cohort Study | 26 years | 19,933 subjects (males, never smokers) | Self-reported | **Exposure:**  History of periodontal disease with bone loss  **Comparison:**  All the remaining participants | Self-reported | **Oropharyngeal cancer*:**  aHR=2.25  (95% CI: 1.30-3.90) |
| Shin et al. (2019) | SouthKorea | Case-Control Study | NR | 424 subjects | Radiographic examination (OPG) | **Exposure:**  Bone loss >3 mm in >2 nonadjacent teeth  **Comparison:**  All the remaining participants | Medical records | **Oral squamous cell carcinoma:**  aOR=3.46  (95% CI: 1.35-8.90) |

**Abbreviations.** a, adjusted; NR, not reported; CI, confidence interval; HR, hazard ratio; OPG, orthopantomogram; OR, odds ratio; USA, United States of America.

**Supplementary Table 2**. Epidemiological evidence linking periodontitis to lung cancer

| **Lung cancer** | | | | | | | | |
| --- | --- | --- | --- | --- | --- | --- | --- | --- |
| **Reference** | **Country** | **Study design** | **Length of follow-up** | **Main sample characteristics** | **Assessment of periodontal status** | **Exposure and comparison** | **Cancer assessment** | **Association with cancer** |
| Michaud et al. (2016) | USA | Prospective Cohort Study | 26 years | 19,933 subjects (males, never smokers) | Self-reported | **Exposure:**  History of periodontal disease with bone loss  **Comparison:**  All the remaining participants | Self-reported | **Lung cancer:**  aHR=0.92  (95% CI: 0.49-1.71) |
| Michaud et al. (2018) | USA | Prospective Cohort Study | 14.7 years (median) | 7,466 subjects | Clinical Examination | **Exposure:**  Severe periodontitis  (CDC/AAP case definition)  **Comparison:**  No periodontitis (CDC/AAP case definition) | Medical records | **Lung cancer:**  aHR=2.37  (95% CI: 1.41-3.99) |
| Wang et al. (2020) | Multinational | Systematic review | 7-41 years (range) | 165,041 subjects | Self-reported (3 studies) and clinical examination (3 studies) | **Exposure:**  Varied according to the included studies  **Comparison:**  Varied according to the included studies | Medical records | **Lung cancer:**  aHR=1.40 (95% CI: 1.25-1.58) |

**Abbreviations.** a, adjusted; AAP, American Academy of Periodontology; CDC, Centers for Disease Control and prevention; CI, confidence interval; HR, hazard ratio; USA, United States of America.

**Supplementary Table 3**. Epidemiological evidence linking periodontitis to breast cancer

| **Breast cancer** | | | | | | | | |
| --- | --- | --- | --- | --- | --- | --- | --- | --- |
| **Reference** | **Country** | **Study design** | **Length of follow-up** | **Main sample characteristics** | **Assessment of periodontal status** | **Exposure and comparison** | **Cancer assessment** | **Association with cancer** |
| Michaud et al. (2018) | USA | Prospective Cohort Study | 14.7 years (median) | 7,466 subjects | Clinical Examination | **Exposure:**  Severe periodontitis  (CDC/AAP case definition)  **Comparison:**  No periodontitis (CDC/AAP case definition) | Medical records | **Breast cancer:**  aHR=0.84  (95% CI: 0.49-1.43) |
| Nwizu et al. (2017) | USA | Prospective Cohort Study | 8.32 years (mean) | 65,869 subjects (postmenopausal women) | Self-reported | **Exposure:**  History of diagnosis from a dentist or dental hygienist of periodontal or gum disease  **Comparison:**  All the remaining participants | Medical records | **Breast cancer:**  aHR= 1.13  (95% CI: 1.03 – 1.23) |
| Shi et al. (2018) | Multinational | Systematic review | 5-18 years (range of median values) | 168,111 subjects | Clinical Examination (2 included studies), NR (6 included studies) | **Exposure:**  Varied according to the included studies  **Comparison:**  Varied according to the included studies | NR | **Breast cancer:**  aRR=1.18  (95% CI: 1.11-1.26) |

**Abbreviations.** a, adjusted; AAP, American Academy of Periodontology; CDC, Centers for Disease Control and prevention; CI, confidence interval; HR, hazard ratio; NR, not reported; RR, risk ratio; USA, United States of America.

**Supplementary Table 4**. Epidemiological evidence linking periodontitis to prostate cancer

| **Prostate cancer** | | | | | | | | |
| --- | --- | --- | --- | --- | --- | --- | --- | --- |
| **Reference** | **Country** | **Study design** | **Length of follow-up** | **Main sample characteristics** | **Assessment of periodontal status** | **Exposure and comparison** | **Cancer assessment** | **Association with cancer** |
| Kim et al. (2020) | South Korea | Retrospective Cohort Study | 10 years | 121,240 subjects | Medical registry | **Exposure:**  Registered at least twice as chronic periodontitis ICD-10 codes (K052-K056)  **Comparison:**  All the remaining participants | Medical records | **Prostate cancer:**  aHR=1.24  (95% CI: 1.16-1.32) |
| Michaud et al. (2016) | USA | Prospective Cohort Study | 26 years | 19,933 subjects (males, never smokers) | Self-reported | **Exposure:**  History of periodontal disease with bone loss  **Comparison:**  All the remaining participants | Self-reported | **Prostate cancer:**  aHR=1.17  (95% CI: 0.94-1.47) |
| Michaud et al. (2018) | USA | Prospective Cohort Study | 14.7 years (median) | 7,466 subjects | Clinical Examination | **Exposure:**  Severe periodontitis  (CDC/AAP case definition)  **Comparison:**  No periodontitis (CDC/AAP case definition) | Medical records | **Prostate cancer:**  aHR=1.10  (95% CI: 0.79-1.54) |

**Abbreviations.** a, adjusted; AAP, American Academy of Periodontology; CDC, Centers for Disease Control and prevention; CI, confidence interval; HR, hazard ratio; ICD, International Classification of Diseases; USA, United States of America.

**Supplementary Table 5**. Epidemiological evidence linking periodontitis to esophageal and gastric cancers

| **Esophageal and gastric cancers** | | | | | | | | |
| --- | --- | --- | --- | --- | --- | --- | --- | --- |
| **Reference** | **Country** | **Study design** | **Length of follow-up** | **Main sample characteristics** | **Assessment of periodontal status** | **Exposure and comparison** | **Cancer assessment** | **Association with cancer** |
| Lo et al. (2021) | USA | 2 Retrospective Cohort Studies | 22-28 years | 148,1444 subjects | Self-reported | **Exposure:**  History of periodontitis  **Comparison:**  All the remaining participants | Medical records | **Esophageal cancer:**  aHR= 1.43  (95% CI: 1.05-1.96)  **Gastric cancer:**  aHR= 1.52  (95% CI: 1.13-2.04) |
| Kim et al. (2022) | SouthKorea | Retrospective Cohort Study | 10 years | 713,201 subjects | Clinical Examination | **Exposure:**  CPI score ≥ 3  **Comparison:**  All the remaining participants | Medical records | **Gastric cancer:**  aHR= 1.14  (95% CI: 1.04-1.24) |
| Nwizu et al. (2017) | USA | Prospective Cohort Study | 8.32 years (mean) | 65,869 subjects (postmenopausal women) | Self-reported | **Exposure:**  History of diagnosis from a dentist or dental hygienist of periodontal or gum disease  **Comparison:**  All the remaining participants | Medical records | **Esophagus cancer:**  aHR= 3.28  (95% CI: 1.64 – 6.53) |

**Abbreviations.** a, adjusted; CI, confidence interval; CPI, community periodontal index; HR, hazard ratio; USA, United States of America.

**Supplementary Table 6**. Epidemiological evidence linking periodontitis to pancreatic cancers

| **Pancreatic cancer** | | | | | | | | |
| --- | --- | --- | --- | --- | --- | --- | --- | --- |
| **Reference** | **Country** | **Study design** | **Length of follow-up** | **Main sample characteristics** | **Assessment of periodontal status** | **Exposure and comparison** | **Cancer assessment** | **Association with cancer** |
| Maisonneuve et al. (2017) | Multinational | Systematic review | 4-41 years  (range) | 302,531 subjects | Self-reported (3 studies), clinical examination (2 studies), medical records (1 study) | **Exposure:**  Varied according to the included studies  **Comparison:**  Varied according to the included studies | NR | **Pancreatic cancer:**  aRR=1.74  (95% CI: 1.41-2.15) |
| Michaud et al. (2007) | USA | Prospective Cohort Study | 16 years | 51,529 subjects (males) | Self-reported | **Exposure:**  History of periodontal disease with bone loss  **Comparison:**  All the remaining participants | Self-reported | **Pancreatic cancer:**  aRR=1.64  (95% CI: 1.19-2.26) |

**Abbreviations.** a, adjusted; CI, confidence interval; CPI, community periodontal index; RR, risk ratio; USA, United States of America.

**Supplementary Table 7**. Epidemiological evidence linking periodontitis to colorectal cancer

| **Colorectal cancer** | | | | | | | | |
| --- | --- | --- | --- | --- | --- | --- | --- | --- |
| **Reference** | **Country** | **Study design** | **Length of follow-up** | **Main sample characteristics** | **Assessment of periodontal status** | **Exposure and comparison** | **Cancer assessment** | **Association with cancer** |
| Kim et al. (2022) | SouthKorea | Retrospective Cohort Study | 10 years | 713,201 subjects | Clinical Examination | **Exposure:**  CPI score ≥ 3  **Comparison:**  All the remaining participants | Medical records | **Colon cancer:**  aHR= 1.13  (95% CI: 1.03-1.24) |
| Li et al. (2021) | Multinational | Systematic review | 6-27 years (range of means) | 337,894 subjects | Self-reported (3 studies), clinical examination (4 studies), medical records (2 studies) | **Exposure:**  Varied according to the included studies  **Comparison:**  Varied according to the included studies | Medical records | **Colorectal cancer:**  aRR= 1.44  (95% CI: 1.18-1.76) |
| Michaud et al. (2016) | USA | Retrospective Cohort Study | 26 years | 19,933 subjects (males, never smokers) | Self-reported | **Exposure:**  History of periodontal disease with bone loss  **Comparison:**  All the remaining participants | Self-reported | **Colorectal cancer:**  aHR=1.03  (95% CI: 0.75-1.39) |
| Michaud et al. (2018) | USA | Retrospective Cohort Study | 14.7 years (median) | 7,466 subjects | Clinical Examination | **Exposure:**  Severe periodontitis  (CDC/AAP case definition)  **Comparison:**  No periodontitis (CDC/AAP case definition) | Medical records | **Colorectal cancer:**  aHR=1.51  (95% CI: 0.90-2.52) |

**Abbreviations.** a, adjusted; AAP, American Academy of Periodontology; CDC, Centers for Disease Control and prevention; CI, confidence interval; CPI, community periodontal index; HR, hazard ratio; RR, risk ratio; USA, United States of America.

**Supplementary Table 8**. Epidemiological evidence linking periodontitis to non-solid malignancies

| **Non-solid malignancies** | | | | | | | | |
| --- | --- | --- | --- | --- | --- | --- | --- | --- |
| **Reference** | **Country** | **Study design** | **Length of follow-up** | **Main sample characteristics** | **Assessment of periodontal status** | **Exposure and comparison** | **Cancer assessment** | **Association with cancer** |
| Bertrand et al. (2017) | USA | Retrospective Cohort Study | 22 years (mean) | 51,529 subjects (males) | Self-reported | **Exposure:**  History of periodontal disease with bone loss  **Comparison:**  All the remaining participants | Self-reported | **Non-Hodgkin lymphoma:**  aHR=1.26  (95% CI: 1.06-1.49)  **Chronic Lymphocytic Leukemia/Small Lymphocytic Lymphomas:**  aHR=1.41 (95% CI: 1.04-1.90) |
| Kim et al. (2022) | South Korea | Retrospective Cohort Study | 10 years | 713,201 subjects | Clinical Examination | **Exposure:**  CPI score ≥ 3  **Comparison:**  All the remaining participants | Medical records | **Hodgkin lymphoma:**  aHR= 0.96  (95% CI: 0.28-3.23)  **Non-Hodgkin lymphoma:**  aHR= 1.05  (95% CI: 0.80-1.36)  **Multiple myeloma:**  aHR= 1.19  (95% CI: 0.75-1.87)  **Leukemia:**  aHR= 1.39  (95% CI: 1.04-1.87) |
| Michaud et al. (2018) | USA | Retrospective Cohort Study | 14.7 years (median) | 7,466 subjects | Clinical Examination | **Exposure:**  Severe periodontitis  (CDC/AAP case definition)  **Comparison:**  No periodontitis (CDC/AAP case definition) | Medical records | **Hematopoietic and lymphatic cancers:**  aHR=0.89  (95% CI: 0.52-1.52) |
| Nwizu et al. (2017) | USA | Retrospective Cohort Study | 8.32 years (mean) | 65,869 subjects (postmenopausal women) | Self-reported | **Exposure:**  History of diagnosis from a dentist or dental hygienist of periodontal or gum disease  **Comparison:**  All the remaining participants | Medical records | **Leukemia:**  aHR= 1.10  (95% CI: 0.83-1.47)  **Non-Hodgkin Lymphoma:**  aHR= 1.08  (95% CI: 0.87-1.34)  **Multiple myeloma: a**HR= 1.05  (95% CI: 0.72-1.54) |

**Abbreviations.** a, adjusted; AAP, American Academy of Periodontology; CDC, Centers for Disease Control and prevention; CI, confidence interval; CPI, community periodontal index; HR, hazard ratio; USA, United States of America.

**Supplementary Table 9**. Epidemiological evidence linking periodontitis to metastasis

| **Metastasis** | | | | | | | | |
| --- | --- | --- | --- | --- | --- | --- | --- | --- |
| **Reference** | **Country** | **Study design** | **Length of follow-up** | **Main sample characteristics** | **Assessment of periodontal status** | **Exposure and comparison** | **Cancer assessment** | **Association with cancer** |
| Kim et al. (2022) | SouthKorea | Retrospective Cohort Study | 10 years | 713,201 subjects | Clinical Examination | **Exposure:**  CPI score ≥ 3  **Comparison:**  All the remaining participants | Medical records | **Metastasis:**  aRR= 1.15  (95% CI: 0.81-1.65) |

**Abbreviations.** a, adjusted; CI, confidence interval; CPI, community periodontal index; RR, risk ratio.
